# Novel Human Adenovirus Strain, Bangladesh

**DOI:** 10.3201/eid1805.111584

**Published:** 2012-05

**Authors:** Yuki Matsushima, Hideaki Shimizu, Atsuko Kano, Etsuko Nakajima, Yoko Ishimaru, Shuvra Kanti Dey, Yuki Watanabe, Fuyuka Adachi, Keiichiro Suzuki, Kohnosuke Mitani, Tsuguto Fujimoto, Tung Gia Phan, Hiroshi Ushijima

**Affiliations:** Kawasaki City Institute of Public Health, Kanagawa, Japan (Y. Matsushima, H. Shimizu, A. Kano, E. Nakajima, Y. Ishimaru);; Nihon University School of Medicine, Tokyo, Japan (K. Dey, H. Ushijima);; Saitama Medical School, Saitama, Japan (Y. Watanabe, F. Adachi, K. Suzuki, K. Mitani);; National Institute of Infectious Diseases, Tokyo (T. Fujimoto);; Blood Systems Research Institute, San Francisco, California, USA (T.G. Phan)

**Keywords:** Adenovirus, viruses, acute gastroenteritis, diarrhea, recombination, Bangladesh

## Abstract

We report a novel human adenovirus D (HAdV-65) isolated from feces of 4 children in Bangladesh who had acute gastroenteritis. Corresponding genes of HAdV-65 were related to a hexon gene of HAdV-10, penton base genes of HAdV-37 and HAdV-58, and a fiber gene of HAdV-9. This novel virus may be a serious threat to public health.

Human adenoviruses (HAdVs) are common pathogens that cause several diseases, such as pneumonia, acute gastroenteritis, and epidemic keratoconjunctivitis (*1*). HAdV infection is also associated with a serious adenovirus syndrome in immunocompromised patients after stem cell transplantation (*2*). Acute gastroenteritis causes illness and death in humans worldwide. Illness is associated with infection of enteric viruses, including rotavirus, astrovirus, norovirus, sapovirus, and adenovirus.

HAdVs are divided into 7 species (HAdV-A–G) on the basis of DNA genome homology. Most acute gastroenteritis related to HAdVs is caused by HAdV-F species (HAdV-40 and HAdV-41 (*3**,**4*). Recently, we detected HAdV-D in feces of children with diarrhea in Bangladesh (*5*). Other HAdV-D strains have also been associated with diarrhea in Kenya and Brazil (*6**,**7*).

We report a novel HAdV-D (HAdV-65) strain detected in feces of 4 children with acute gastroenteritis during October 2004–March 2005 in Bangladesh (*5*) and results of hexon, penton base, and fiber gene sequence analyses. We also report the full genome sequence of this virus, whose corresponding genes are closely related to the hexon gene of HAdV-10, penton base genes of HAdV-37 and HAdV-58, and the fiber gene of HAdV-9.

## The Study

Cloned virus (3 plaque purifications) was propagated in an A549 cell line, which was maintained in minimal essential medium supplemented with 1% fetal bovine serum (Cansera International Inc., Toronto, Ontario, Canada). Cultures were observed for 3–4 weeks for a cytopathic effect. After a cytopathic effect was observed, cell lysates were centrifuged at 1,430 × *g* for 20 min at 4°C. Supernatants were centrifuged at 72,000 × *g* for 3 h at 4°C. Pellets were resuspended in sterile water and treated with 10 µL (20 mg/mL) of proteinase K. DNA was extracted by using the phenol:chloroform:isoamyl alcohol (25:24:1) method (Invitrogen, Carlsbad, CA, USA) and precipitated with isopropyl alcohol.

PCR was performed in a total volume of 50 μL containing 20 pmol/μL of each primer, 2.5 mmol/L of dNTP, 1.25 units of GXL DNA polymerase (Takara, Shiga, Japan), and 5 μL of DNA template. After PCR products were purified by using the MinElute PCR Purification Kit (QIAGEN, Hilden, Germany), cycle sequencing was conducted by using the Genome Lab DTCS Quick Start Kit (Beckman Coulter Inc., Fullerton, CA, USA).

The complete genome of HAdV-65 was sequenced by using the primer walking method. The 5′ terminus of full-length DNA was phosphorylated with 20 units of T4 polynucleotide kinase and 0.5 μL of 100 mmol/L ATP, and ligated to a blunt *Eco*RI-*Not*I-*Bam*HI adaptor (1 pmol/μL) for 3 h at 8°C by using the DNA Ligation Mighty Mix Kit (Takara), PCRs with primer pairs containing adaptor sequences were conducted as described (*8*). DNA sequences were assembled by using the CEQ 2000XL DNA Analysis System version 4.3.9 (Beckman Coulter Inc.).

The genome of HAdV-65 was 35,172 bp. It had a GC content of 56.9%, and the inverted terminal repeat sequence of this virus was 150 bp. Phylogenetic trees were generated by using the maximum-likelihood method with MEGA5 (www.megasoftware.net) after alignment was performed by using ClustalW (www.clustal.org).

**Figure A1:**
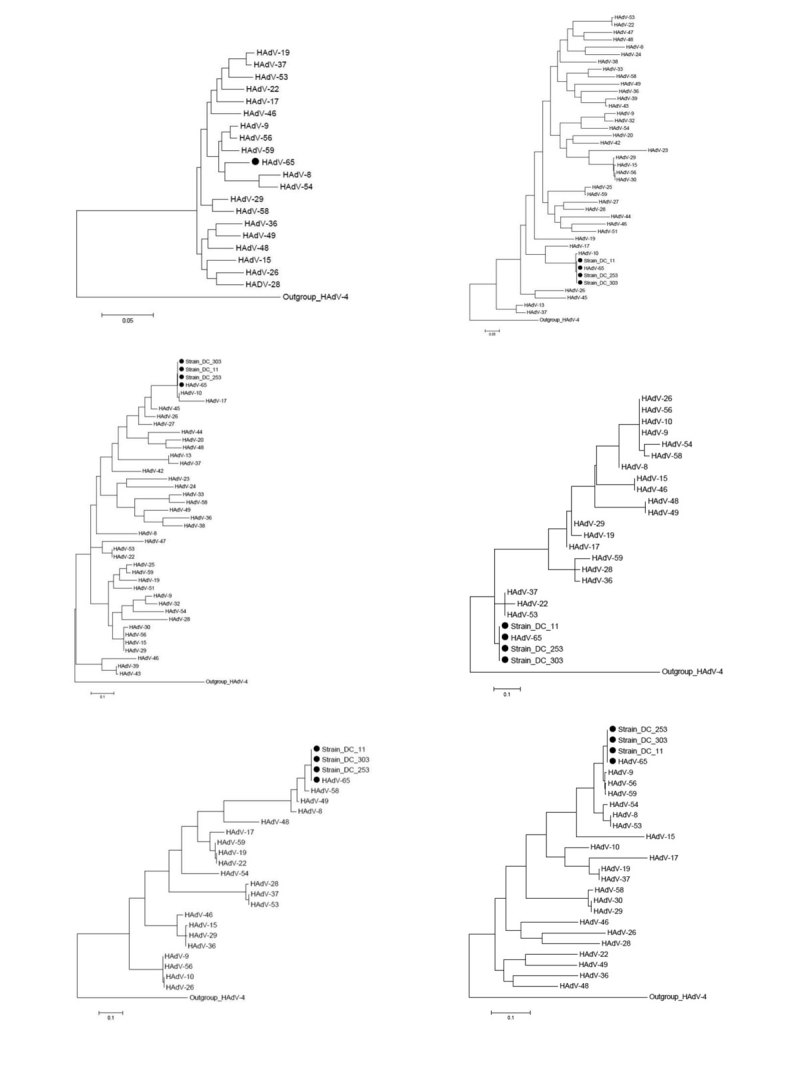
Phylogenetic trees of a novel human adenovirus (HAdV-65), 3 strains (DC 11, DC 253, and DC 303), and other HAdV-D reference strains. Full genome sequences (except for the 3 strains) (A), loop 1 (B) and loop 2 (C) sequences of hexon gene, hypervariable loop 1 of penton base gene (D), Arg-Gly-Asp (RGD) loop of penton base gene (E), and fiber gene (F). Black dots indicate new strains isolated in this study. Scale bars indicate nucleotide substitutions per site. Adenoviruses HAdV-8 (DQ149614, AB448767), HAdV-9 (AJ854486), HAdV-10 (DQ149615, DQ375463, AB369368), HAdV-13 (DQ149616), HAdV-15 (DQ149617, AB562586), HAdV-17 (AF108105), HAdV-19 (DQ149618, EF121005), HAdV-20 (DQ149619), HAdV-22 (DQ149620, FJ404771), HAdV-23 (DQ149621), HAdV-24 (DQ149622), HAdV-25 (DQ149623), HAdV-26 (DQ140624, EF153474), HAdV-27 (DQ149625), HAdV-28 (DQ149626, FJ824826), HAdV-29 (DQ149627, AB562587), HAdV-30 (DQ149628, AF447393), HAdV-32 (DQ149629), HAdV-D33 (DQ149630), HAdV-36 (DQ149631, GQ384080), HAdV-37 (DQ149632, DQ900900), HAdV-38 (DQ149633), HAdV-39 (DQ149634), HAdV-42 (DQ149635), HAdV-43 (DQ149636), HAdV-44 (DQ149637), HAdV-45 (DQ149638), HAdV-46 (DQ149639, AY875648), HAdV-47 (DQ149640), HAdV-48 (EF153473), HAdV-49 (DQ149641, DQ393829), HAdV-51 (DQ149642), HAdV-53 (FJ169625), HAdV-54 (NC012959), HAdV-56 (HM770721), HAdV-58 (HQ883276), HAdV-59 (JF799911), and HAdV-4 (AY487947) were used as reference strains.

Complete genome analysis showed that HAdV-65 was >5.0% distant from any other HAdV-D reference strains (Figure A1, panel A). On the basis of hypervariable loop 1 and loop 2, which encode the neutralization epitope of HAdVs, this virus clustered with HAdV-10 (Figure A1, panels B and C). However, HAdV-65 was closely related to HAdV-37 and HAdV-58 in the hypervariable loop 1 and the Arg-Gly-Asp (RGD) loop of the penton base gene, respectively (Figure A1, panels D and E). Phylogenetic analysis also showed that this novel virus clustered with HAdV-9 on the basis of the fiber gene sequence (Figure A1, panel F). Sequences of HAdV-65 and 3 other strains (DC 11, 253, and 303) isolated from infants in this study had identical hexon, penton base, and fiber genes.

**Figure A2:**
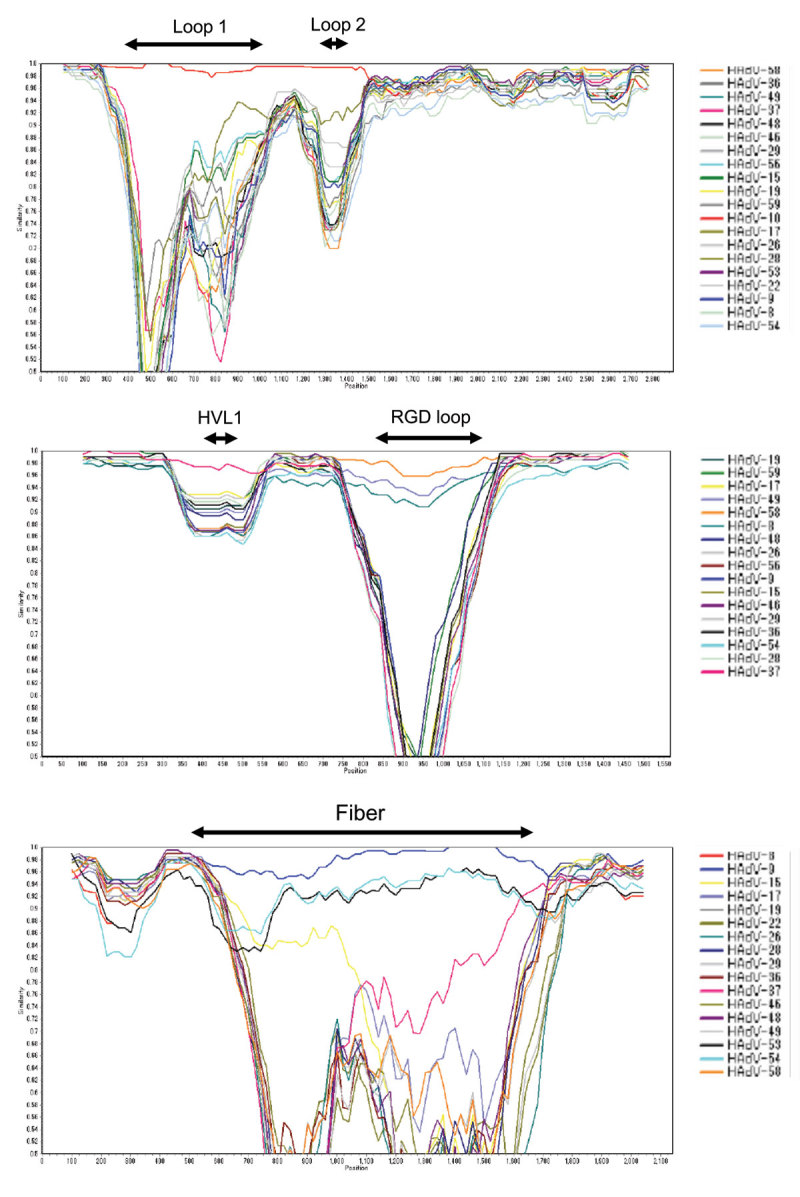
SimPlot analyses for identifying recombination sites in the hexon gene (A), penton base gene (B), and fiber gene, including the outside of the coding sequence, (C) of human adenoviruses (HAdVs). The x-axes indicate nucleotide positions in these genes, and the y-axes indicate similarities between HAdV-65 and other HAdV-D reference strains. Parameters used were a 200-bp window, a 20-bp step, and a Kimura 2-parameter. HVL1; hypervariable loop 1; RGD, Arg-Gly-Asp.

Potential recombination in HAdV-65 was investigated by using the SimPlot program (http://sray.med.som.jhmi.edu/RaySoft/simplot_old/Version1/SimPlot_Doc_v13.html). DNA sequence alignments were created by using DNASIS Pro (Hitachi Solutions, Tokyo, Japan). SimPlot analysis showed no potential recombination in the hexon and fiber genes (Figure A2, panels A and C), and recombination between the hypervariable loop 1 and the RGD loop was predicted in the penton base gene (Figure A2, panel B). HAdV-65 had nucleotide identities of 97.9% to HAdV-10 in the hexon gene, 92.3% and 96.7% to HAdV-37 and HAdV-58, respectively, in the penton base gene, and 98.2% to HAdV-9 in the fiber gene. The GenBank accession number for HAdV-65 is AP012285.

## Conclusions

We report the complete genome of HAdV-65, a novel human adenovirus isolated from children with gastroenteritis. Recombination is an essential feature for viral evolution and immune escape. Recombination can be facilitated by antiviral immune pressure and co-infection with different HAdV strains of the same species (*9*). Recently, newly identified HAdVs appeared to originate by recombination among >2 viruses. HAdV-53 was reported as a novel recombinant HAdV with a close genetic relationship to loop 1 and loop 2 of HAdV-22, the penton base gene of HAdV-37, and the fiber gene of HAdV-8. HAdV-56 had a loop 1 and loop 2 highly similar to those of HAdV-15 (*10**,**11*). All other regions of the genome were genetically related to HAdV-9.

HAdV-58 was recently characterized as a novel HAdV with unique hexon and fiber genes of HAdV-25 and HAdV-29 (*12*). In the present study, we demonstrated that hexon and fiber coding regions of HAdV-65 were formed by recombination in regions around these genes but not by potential recombination within these genes. The potential recombination site within the penton base gene is located at the central position between hypervariable loop 1 and the RGD loop.

These findings suggest that the most conserved sequences around the hexon and fiber genes and in the penton base gene may play a major role in recombination. In addition, this recombination mechanism may be more efficient in enabling new processes of infection and immune escape for maintaining HAdVs than individual small mutations, such as insertions, substitutions, and deletions.

Most recombinant HAdVs have been found in AIDS patients (*9**,**13*). The RGD loop of the penton base protein can be digested by trypsin secreted in the intestines, which results in inhibition of proliferation of HAdV except for HAdV-F types in the intestines (*14*). In this study, HAdV-65 was isolated from infants who had lower immunocompetence and secretion rates of digestive enzymes than adults. These results indicate that emergence of HAdV-65 might have been caused by long coexistence of multiple HAdV-D types and depending on a decrease in immunity, as observed in AIDS patients, and decreased digestive capacity in the intestines.

We detected this type of recombination not only in HAdV-65, but also in 3 other HAdV strains that had a genome sequence identical with that of HAdV-65 from children in Bangladesh during 2004–2005. This finding indicates that this virus might be a newly emerging HAdV, which might be a serious threat to public health.

## References

[1] Benko M. Adenoviruses: pathogenesis. In: Mahy BW, van Regenmortel MH, editors. Encyclopedia of virology. 3rd ed. Oxford: Elsevier; 2008. p. 24–9.

[2] Kampmann B, Cubitt D, Walls T, Naik P, Depala M, Samarasinghe S, Improved outcome for children with disseminated adenoviral infection following allogeneic stem cell transplantation. Br J Haematol. 2005;130:595–603. 10.1111/j.1365-2141.2005.05649.x 16098075

[3] Akihara S, Phan TG, Nguyen TA, Hansman G, Okitsu S, Ushijima H, Existence of multiple outbreaks of viral gastroenteritis among infants in a day care center in Japan. Arch Virol. 2005;150:2061–75. 10.1007/s00705-005-0540-y 15841336

[4] Shimizu H, Phan TG, Nisimura S, Okitsu S, Maneekarn N, Ushijima H, An outbreak of adenovirus serotype 41 infection in infants and children with acute gastroenteritis in Maizuru City, Japan. Infect Genet Evol. 2007;7:279–84. 10.1016/j.meegid.2006.11.005 17157081

[5] Dey SK, Shimizu H, Phan TG, Hayakawa Y, Islam A, Salim AFM, Molecular epidemiology of adenovirus infection among infants and children with acute gastroenteritis in Dhaka City, Bangladesh. Infect Genet Evol. 2009;9:518–22. 10.1016/j.meegid.2009.02.001 19460318

[6] Filho EP, da Costa Faria NR, Fialho AM, de Assis RS, Almeida MMS, Rocha M, Adenoviruses associated with acute gastroenteritis in hospitalized and community children up to 5 years old in Rio de Janeiro and Salvador, Brazil. J Med Microbiol. 2007;56:313–9. 10.1099/jmm.0.46685-0 17314359

[7] Magwalivha M, Wolfaardt M, Kiulia NM, van Zyl WB, Mwenda JM, Taylor MB, High prevalence of species D human adenoviruses in fecal specimens from urban Kenyan children with diarrhea. J Med Virol. 2010;82:77–84. 10.1002/jmv.21673 19950234

[8] Matsushima Y, Shimizu H, Phan TG, Ushijima H. Genomic characterization of a novel human adenovirus type 31 recombinant in the hexon gene. J Gen Virol. 2011;92:2770–5. 10.1099/vir.0.034744-0 21880842

[9] Robinson CM, Seto D, Jones MS, Dyer DW, Chodosh J. Molecular evolution of human species D adenoviruses. Infect Genet Evol. 2011;11:1208–17. 10.1016/j.meegid.2011.04.031 21570490 PMC3139803

[10] Walsh MP, Chintakuntlawar A, Robinson CM, Madisch I, Harrach B, Hudson NR, Evidence of molecular evolution driven by recombination events influencing tropism in a novel human adenovirus that causes epidemic keratoconjunctivitis. PLoS ONE. 2009;4:e5635–48. 10.1371/journal.pone.0005635 19492050 PMC2685984

[11] Robinson CM, Singh G, Henquell C, Walsh MP, Peigue-Lafeuille H, Seto D, Computational analysis and identification of an emergent human adenovirus pathogen implicated in a respiratory fatality. Virology. 2011;409:141–7. 10.1016/j.virol.2010.10.020 21056888 PMC3006489

[12] Liu EB, Ferreyra L, Fischer SL, Pavan JV, Nates SV, Hudson NR, Genetic analysis of a novel human adenovirus with a serologically unique hexon and a recombinant fiber gene. PLoS ONE. 2011;6:e24491–501. 10.1371/journal.pone.0024491 21915339 PMC3168504

[13] Crawford-Miksza LK, Schnurr DP. Adenovirus serotype evolution is driven by illegitimate recombination in the hypervariable regions of the hexon protein. Virology. 1996;224:357–67. 10.1006/viro.1996.0543 8874497

[14] Albinsson B, Kidd AH. Adenovirus type 41 lacks an RGD αv-integrin binding motif on the penton base and undergoes delayed uptake in A549 cells. Virus Res. 1999;64:125–36. 10.1016/S0168-1702(99)00087-8 10518709

